# A Preliminary Prospective Study on the Association of Polymorphisms in *BDNF*, *ARRB2* and *KCNJ6* and Response to Fentanyl for Pain Management in Advanced Cancer

**DOI:** 10.1177/10732748251372675

**Published:** 2025-09-19

**Authors:** John Prenzler, Alison Haywood, Heidi Sutherland G, Omar Ibrahim, Matthew Zunk, Sudeep Bista R, Phillip Good, Lyn Griffiths R, Janet Hardy, Larisa M Haupt

**Affiliations:** 1 School of Pharmacy and Medical Sciences, Griffith University, Gold Coast, QLD, Australia; 2 Mater Research Institute, University of Queensland, Brisbane, QLD, Australia; 3 Centre for Genomics and Personalised Health, Genomics Research Centre, School of Biomedical Sciences, Queensland University of Technology, Brisbane, QLD, Australia; 4 Department of Palliative Care, St Vincent’s Private Hospital, Brisbane, QLD, Australia; 5 ARC Training Centre for Cell and Tissue Engineering Technologies, Queensland University of Technology, Brisbane, QLD, Australia; 6 Max Planck Queensland Centre for the Materials Sciences of Extracellular Matrices, Brisbane, QLD, Australia

**Keywords:** palliative care, opioids, fentanyl, KCNJ6, ARRB2, BDNF

## Abstract

**Introduction:**

Pain is common in cancer patients, particularly in the advanced stage of the disease with more than 70% prevalence. Despite research regarding cancer pain management, clinicians’ ability to predict and manage patient pain remains a significant challenge.

**Patients and Methods:**

This sub-study of a prospective, open label, dose individualization study, investigated how selected single nucleotide polymorphisms (SNPs) in the *KCNJ6, ARRB2, and BDNF* genes may affect fentanyl dose requirements and response. Fifty-six adult inpatients or outpatients of oncology and pallateive care services who met the eligibility criteria were recruited Administration of transdermal fentanyl was monitored, and participant characteristics (age, height, weight, type of cancer, liver and renal function, fentanyl dose) and pain scores (numerical rating scale) recorded. SNP genotyping was conducted using pyrosequencing (*KCNJ6* and *BDNF*) and TaqMan assays (*ARRB2*). Statistical analysis included patient characteristics, observed and expected genotype frequencies, genotype and fentanyl dose/pain score, along with categories of low (≤3.0/10) or high (>3.0/10) pain scores and low (≤50 mcg/hr) or high fentanyl doses (>50 mcg/hr).

**Results:**

The median fentanyl dose administered was 50 mcg/hr, with a range of 12 to 300 mcg/hr, with the mean pain score 3.0/10.0 (SD:2.3). No association was found between patient characteristics, fentanyl dose, and pain score (*P*-values >0.05) in the European and Asian population. No association was found for *KCNJ6* (rs2070995), *ARRB2* (rs34230287, rs3786047, rs1045280, rs2036657), and *BDNF* (rs7934165, rs10835210, rs1491850) in relation to dose and pain score.

**Conclusion:**

These results may show no association between the SNPs examined in *KCNJ6*, *ARRB2,* and *BDNF* with fentanyl dose or response in the population of the study, as this was the case for the subgroups of the population we were able to divide individuals in based on allele groups. This evidence may enhance existing studies, driving the ongoing advancement and refinement of gene-drug dosing guidelines.

## Plain Language Summary

Pain is common in cancer patients, particularly in the advanced stage of the disease. Despite much research, the ability of clinicians to predict and manage patient pain remains a significant challenge. We investigated whether genetic variables can explain patient response to fentanyl. Previous studies have identifed an observed variability in response to fentanyl dose between patients. Here we sought to determine relationships between genetic variants. Our results showed no association between the SNPs examined in KCNJ6, ARRB2 and BDNF with fentanyl dose or response in the population of the study. However, this evidence has enhanced existing studies, driving the ongoing advancement and refinement of gene-drug dosing guidelines.

## Introduction

Genetic variables, termed Single Nucleotide Polymorphisms (SNPs), are mutations that occur in a minority of individuals and have the potential to influence their susceptibility to certain diseases or affect how the body responds to or metabolizes medicines. This study investigated SNPs in three genes, *KCNJ6*, *ARRB2* and *BDNF*, for their association with patient opioid response. With an observed variability in response to dose, determining the relationship between SNPs and response to fentanyl in patients with advanced cancer could lead to an improvement in the effective use of medicines and quality of life through personalized pain management.^1-3^

Variants in the *KCNJ6* gene (A1032 G and G1250 A) have been identified to play a role in the breakdown of biologically active catecholamines, such as dopamine, noradrenaline, and adrenaline, mediator of physiological processes including pain modulation.^4^ A previous study suggested a potential relationship between the rs2070995 AA genotype in *KCNJ6* and opioid dosing requirements in Japanese chronic pain patients.^5^ *KCNJ6* encodes potassium inwardly rectifying channels (Kir3.2, GIRK2), which are essential for opioid receptor transmission.^6^ Both *KCNJ3 *(GIRK1) and *KCNJ6* (GIRK2) genes have been shown to affect pain and opioid analgesic responses in animal studies.^6^ GIRK channels are activated by G-proteins after opioid receptor stimulation, leading to the efflux of potassium ions, hyperpolarization of membrane potential, and reduction of nociceptive transmission. In patients undergoing major abdominal surgery, homozygous carriers of the A allele of the A1032 G SNP were found to require rescue pain medication more frequently.^6^ However, no association with postsurgical acute pain ratings were observed. The relationship of *KCNJ6* to opioid response has to date yielded mixed results, indicating the need for further study due to the variations in prior research findings and the functional consequence of these variants on mRNA and protein expression.

β-arrestin2 (*ARRB2*) is a critical regulatory protein involved in the desensitization and internalization of receptors within the G-protein-coupled receptor (GPCR) superfamily.^7^ Polymorphisms in the *ARRB2* gene have shown a positive correlation with morphine response; however, their association with fentanyl treatment response remains unknown.^8^ Notably, the CASP1 rs554344 homozygous variant genotype and *ARRB2* variant diplotype have been found to be associated with an increased risk of adverse events. In contrast, the *TGFB1* rs1800469 homozygous wildtype genotype was linked to a decreased occurrence of adverse events.^9^

Brain-derived neurotrophic factor (BDNF) is a key neurotrophic factor expressed in the hippocampus and cerebral cortex. BDNF promotes neuronal growth, synaptic plasticity, and cognitive processes including learning and memory.^10^ The rs6265 polymorphism in the *BDNF* gene alters the intracellular processing of *BDNF*, affecting its secretion.^11^ *BDNF* also influences neurotransmitters such as serotonin and dopamine, impacting the body reward system.^11^ This polymorphism is associated with a decreased risk of alcohol dependence, while the homozygous wild-type genotype increases susceptibility to alcohol addiction.^11^

This study was comprised of patients with advanced malignant disease with frailty, and poor performance status. Pain, which worsens as disease progresses, is commonly managed with opioids, adjuvant analgesics, and physical therapies.^12^ Oral opioids are preferred, but other administration routes (subcutaneous, rectal, spinal) are also used. Based on current guidelines transdermal fentanyl is to be considered for pain relief in moderate to severe cancer pain.^12-15^ In addition pain remains often poorly managed or undertreated in cancer patients demonstrating the need for personalized approaches to pain management.^16^ Fentanyl, a potent opioid, is used in transdermal and transmucosal forms for cancer pain.^17^ Transmucosal formulations target breakthrough pain, while the transdermal patch is preferred for patients with high pill burden or gastrointestinal side effects.^17^ Fentanyl is a highly lipophilic drug, acting as a full agonist at the mu-opioid receptor and is estimated to be 50-100 times more potent than morphine.^18^

Current Clinical Pharmacogenetics Implementation Consortium (CPIC) guidelines exist to guide dosing based on genetic profile for several genes (CYP2D6, OPRM1, and COMT), with evidence suggesting CYP2D6 SNPs influence dosing requirements of opioids in patients.^15^ Based on the role that *KCNJ6*, *BDNF*, and *ARRB2* have regarding opioids, this study aimed to investigate these genes to provide further data to aid in developing a reliable clinical tool to aid in managing patient dosing of fentanyl. Although disease progression is relatively minor in advanced stages, it is essential to recognize that pain is subjective and varies among individuals.^16^ In addition pain remains often poorly managed or undertreated in cancer patients demonstrating the need for personalized approaches to pain management.^16^ This study was designed to investigate whether genetic variables can explain patient response to fentanyl.

## Patients and Methods

### Study Participants and Procedures

Adult in-patients or outpatients of the oncology and palliative care services of Mater Adults Hospital in Brisbane were eligible for inclusion in a prospective, open label, dose individualization study on the use of fentanyl for pain management in advanced cancer at end of life.^5^ A secondary aim of this study was to determine the association of SNPs in *BDNF*, *ARRB2* and *KCNJ6* and fentanyl requirements for pain management. Patients aged >18 years, with a diagnosis of malignant disease, receiving fentanyl via a transdermal patch, and able to provide written consent and blood samples, were eligible for inclusion in the study. Ethics approval was granted by the Human Research Ethics Committees at Mater Health Services (# HREC/1909A) and Griffith University (# PHM/16/13/HREC). Patients were excluded if they were using fentanyl for breakthrough analgesia. Patients presenting with oral mucositis, active oral infections, or xerostomia were excluded if these conditions rendered saliva collection either painful or impractical. Fentanyl was administered via the transdermal route (Durogesic, Janssen-Cilag, Australia) with the dose titrated according to clinical need by the palliative care specialists, taking into consideration any current breakthrough opioids. Participant characteristics, including type of cancer, liver and renal function, fentanyl dose and pain score, were recorded. Participants rated their pain on a numerical rating scale from 0 to 10, with a score of 0 representing “no pain” and 10 representing “pain as bad as you can imagine”, using the Brief Pain Inventory.^19^ Pain scores were recorded each time blood and saliva were collected and at a time convenient to the participant. The reporting of this study conforms to STROBE guidelines.^20^

### DNA Isolation and Genotyping

Genomic DNA (gDNA) was extracted from whole blood collected into EDTA tubes using an in-house salting-out method [5]^21^ at the Genomics Research Centre, Queensland University of Technology, Brisbane. A NanoDropTM ND-1000 spectrophotometer (ThermoFischer Scientific Inc, Waltham, MA, USA) was used to measure DNA concentration and purity before dilution to 15-20 ng/mL and storing as gDNA stock at 4°C. Genotyping of *KCNJ6* (rs2070995) and *BDNF* (rs1491850, rs7934165, rs10835210) was conducted via pyrosequencing with primers designed using Pyromark Assay Design software (QIAGEN). Pyrosequencing was performed on a QSeq platform (BioMolecular Systems) using Pyromark Gold Q24 reagents (QIAGEN). Sequencing traces were analyzed with Qseq software, version 2.1.3 (BioMolecular Systems). *ARRB2* was genotyped using TaqMan® SNP Genotyping Assays from Applied Biosystems (Life Technologies, Carlsband, CA, USA) specifically designed for amplification and genotype identification of each SNP (rs34230287: C_60483877_10, rs3786047: C_27500850_10, rs1045280_20: C_8718195_20, rs2036657: C_11954713_10). The final optimized PCR reaction conditions consisted of 1 μL of DNA (20 ng/ μL concentration), 2.5 μL of Taqman® Universal PCR Master Mix (2X) (ThermoFischer Scientific, Catalog number 4364338), 0.25 μL of SNP genotyping assay probe-primer mix (20X), and 1.25 μL of nuclease free water in each 5 μL reaction volume. The PCR thermocycling conditions consisted of one cycle at 95°C for 10 min, followed by 40 cycles at 95°C for 15 s and 60°C for 1 min. Sanger sequencing of a subset of samples was performed on an ABI3500 (Life Technologies, Carlsband, CA, USA) to confirm the genotypes for each SNP. All genotyping was conducted by investigators blinded to sample identity.

### Statistical Analysis

Data was analyzed using IBM SPSS Statistics for Windows, version 26.0 (Armonk, NY: IBM Corp). Clinical data are described as mean ± standard deviation (SD) or medians and interquartile ranges (IQR), as appropriate for continuous measures. The adequacy of each statistical test was assessed by examining residuals for heterogeneity and normality. Deviation of Hardy-Weinberg equilibrium (HWE) was determined by comparing the observed genotype frequencies with the expected values using the chi-square (χ^2^) test. The Kruskal-Wallis H test was used to determine whether genotypes were associated with fentanyl dose or pain score.

Chi-square analysis was used to determine significant associations for high pain score (>3/10) and high fentanyl dose (>50 mcg/day) when outcomes were categorized. Significance was considered if *P* < 0.05. Regression analysis was used to examine whether outcomes depended on non-genetic patient characteristics. The observed minor allele frequency (MAF) was compared to the MAF for relevant populations reported for ALFA and 1000Genomes in the dbSNP (National Center for Biotechnology Information).^22^

A sample size of 50 participants, providing two to four samples, was determined to be the minimum number necessary to generate satisfactory estimates of the structural parameters (clearance and volume of distribution) and the variance parameters (interindividual and inter-occasion variability) for non-linear mixed effect modelling (population pharmacokinetic modelling) for the dose individualization study.

## Results

### Patient Characteristics

**Figure 1.**
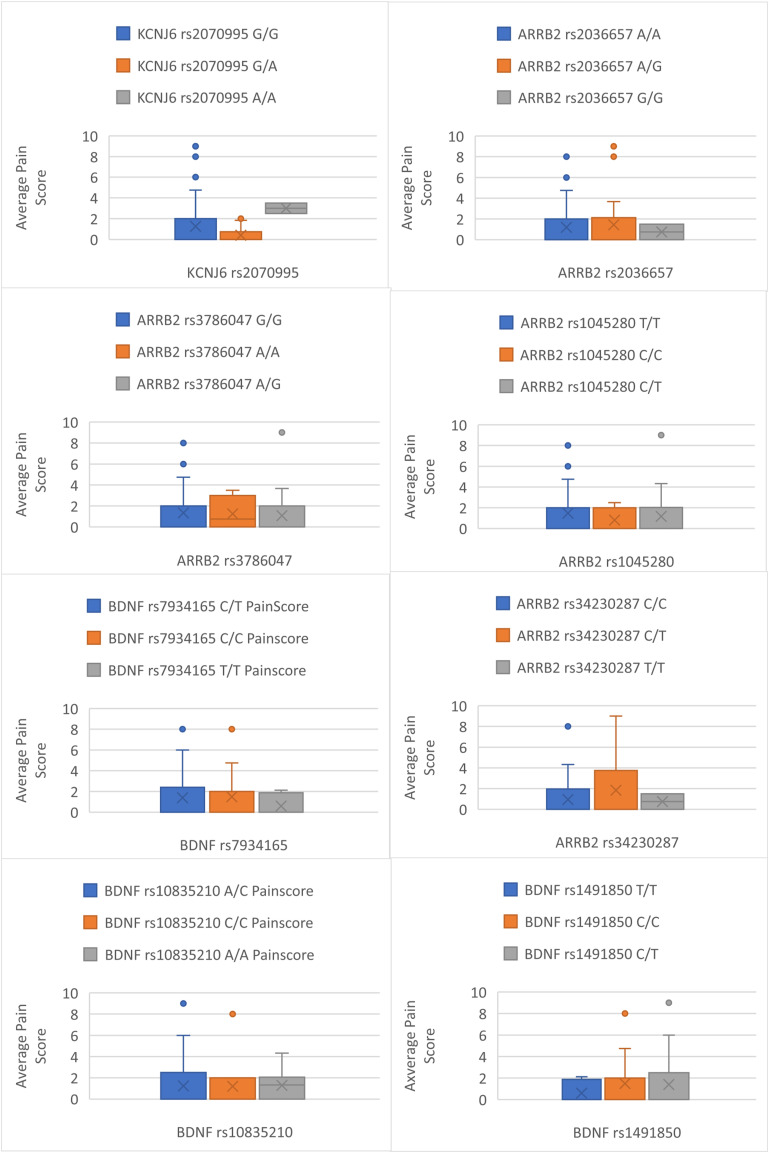
Box Plots Detailing Differences Between Genotypes Average Pain Score

**Figure 2.**
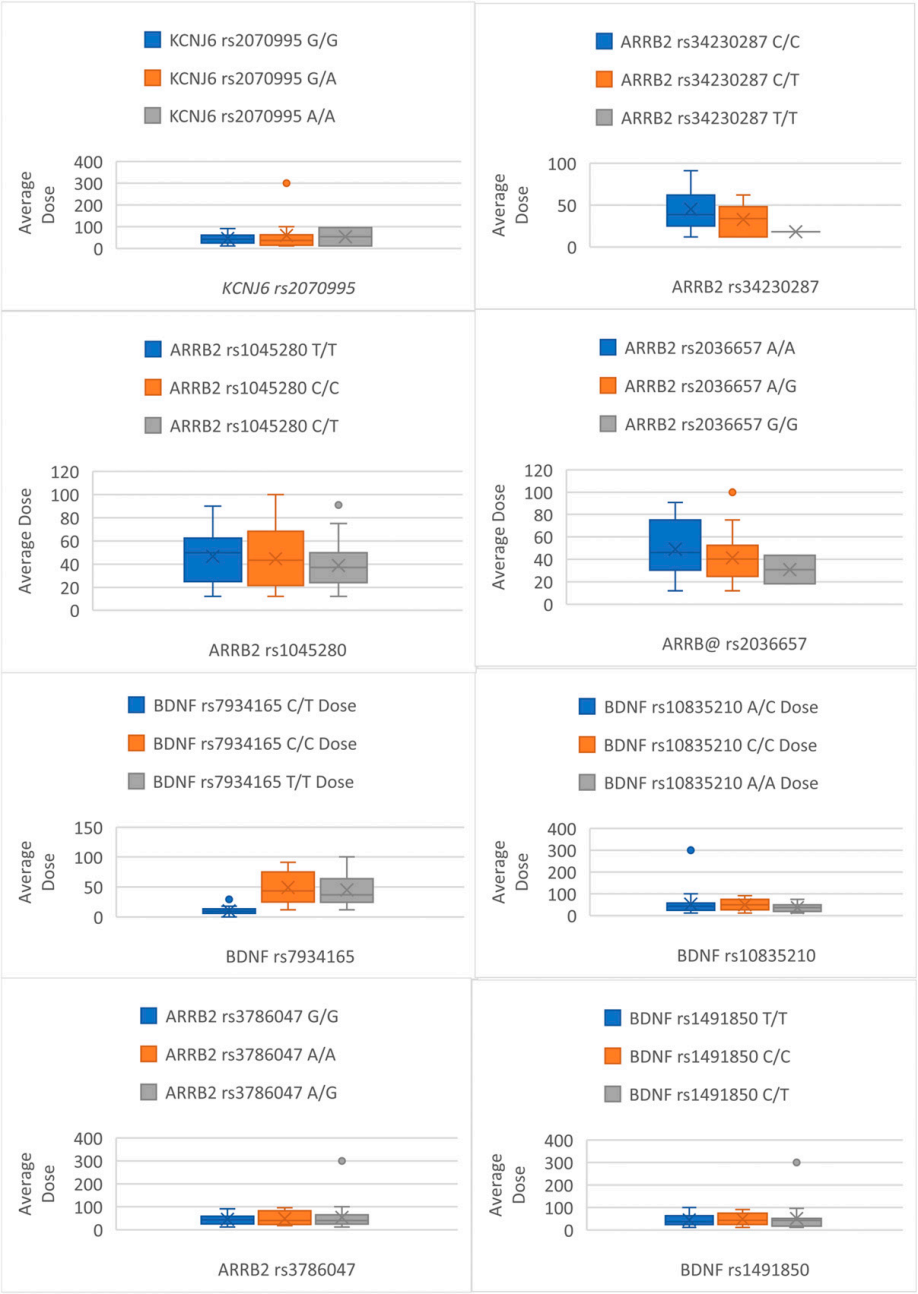
Box Plots Detailing Differences Between Genotypes Average Dose

Complete pain scores and genotyping data were available for all 56 participants. The median (IQR) age, weight, and body mass index (BMI) were 69.5 ± 16 years, 70.0 ± 20.1 kg and 24.9 ± 8.0 kg/m^2^, respectively, with 34 (60.7%) patients being male. The most common cancer types included 8 (14.2%) breast, 8 (14.2%) lung, 6 (10.7%) ovarian, and 5 (8.9%) prostate cancers. The prescribed dose of fentanyl ranged from 12-300 mcg. The median (IQR) number of samples obtained from each participant was 2 ± 3. The fentanyl dose was titrated as required to control pain. For participants providing multiple samples, fentanyl dose and pain scores were averaged across all samples (Figures 1 and 2). None of the patient characteristics, including gender, age, height, weight, BMI, renal and liver function, significantly determined the outcomes of fentanyl dose or pain score (Table 1). The median (IQR) fentanyl dose was 42.9 ± 36.5 mcg, and the patient’s reported pain score was 2/10 ± 2.8.

**Table 1.** Regression Analysis of the Total Population When Taken Into Account Considering Each Fentanyl Dosing or Pain Score Reading, *P* Value: <0.5 = Statistically Significant >0.5 not Statistically Significant

Patient characteristics	Fentanyl dose (*P* value)	Pain score (*P* value)
Age	0.07	0.145
Gender	0.46	0.372
Cancer diagnosis	0.745	0.650
BMI	0.858	0.964
BSA	0.896	0.953
Weight (kg)	0.673	0.072
Liver function		
AST	0.700	0.164
ALT	0..943	0.204
ALP	0.324	0.577
LDH	0.705	0.610
GGT	0.331	0.607
Kidney function		
Creat	0.140	0.740
ALB	0.911	0.710
UREA	0.787	0.080
eGFR	0.930	0.143
Creat CL	0.940	0.120
Blood cell counts		
HB	0.432	0.534
WBC	0.708	0.629
PLTS	0.432	0.077
Age (mean, range, standard deviation)	67, (39-90), 11.148	
Gender (n, %)	M = 22 (39.3%) F = 34 (60.7%)	
Fentanyl dose (mcg) (Median, range, standard deviation)	50, (12-300), 41.97	
Pain score average (mean, range, standard deviation)	3, (0-10), 2.26	
Weight (kg) (Mean, range, standard deviation)	69.86, (41.8-110), 20.67	
BMI (kg/m^2^) (Mean, range, standard deviation)	25.07, (14.99-41.87), 7.64	

### Association of Polymorphisms in KCNJ6 and Fentanyl Response

**Figure 3.**
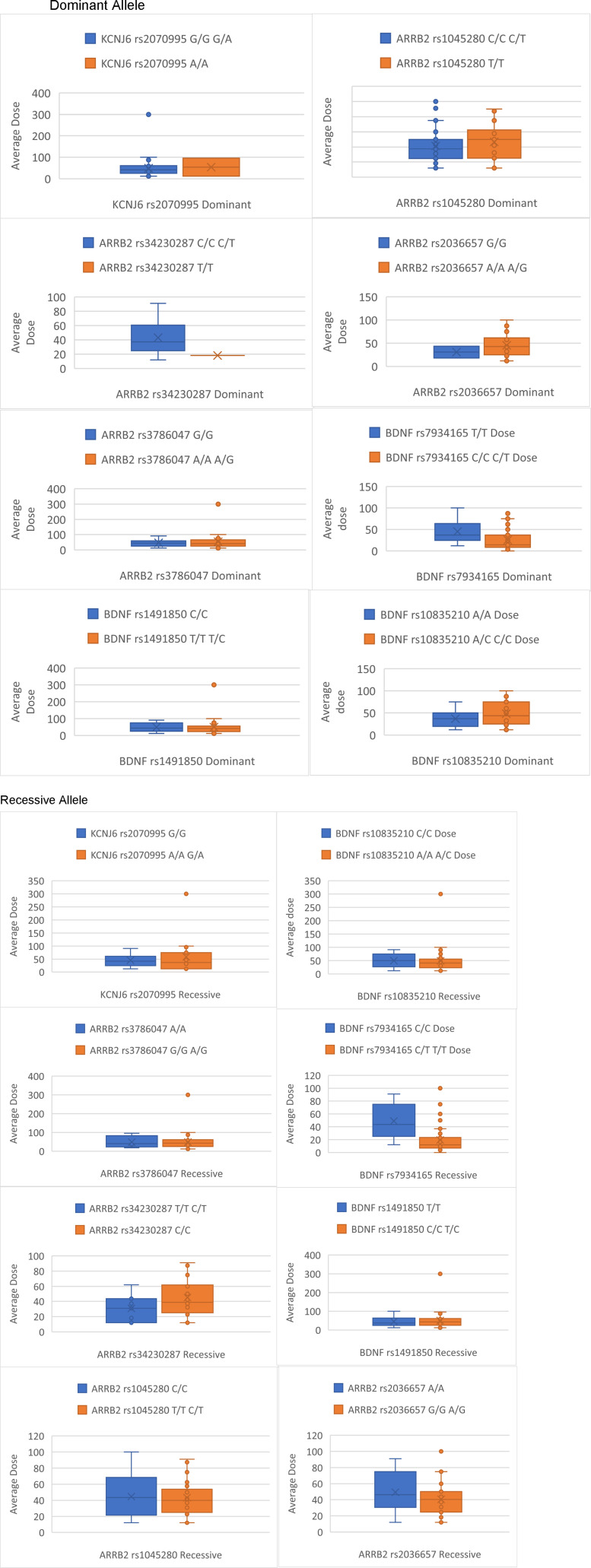
Box Plots Detailing Differences Between Dominant and Recessive Genotypes Average Dose

**Figure 4.**
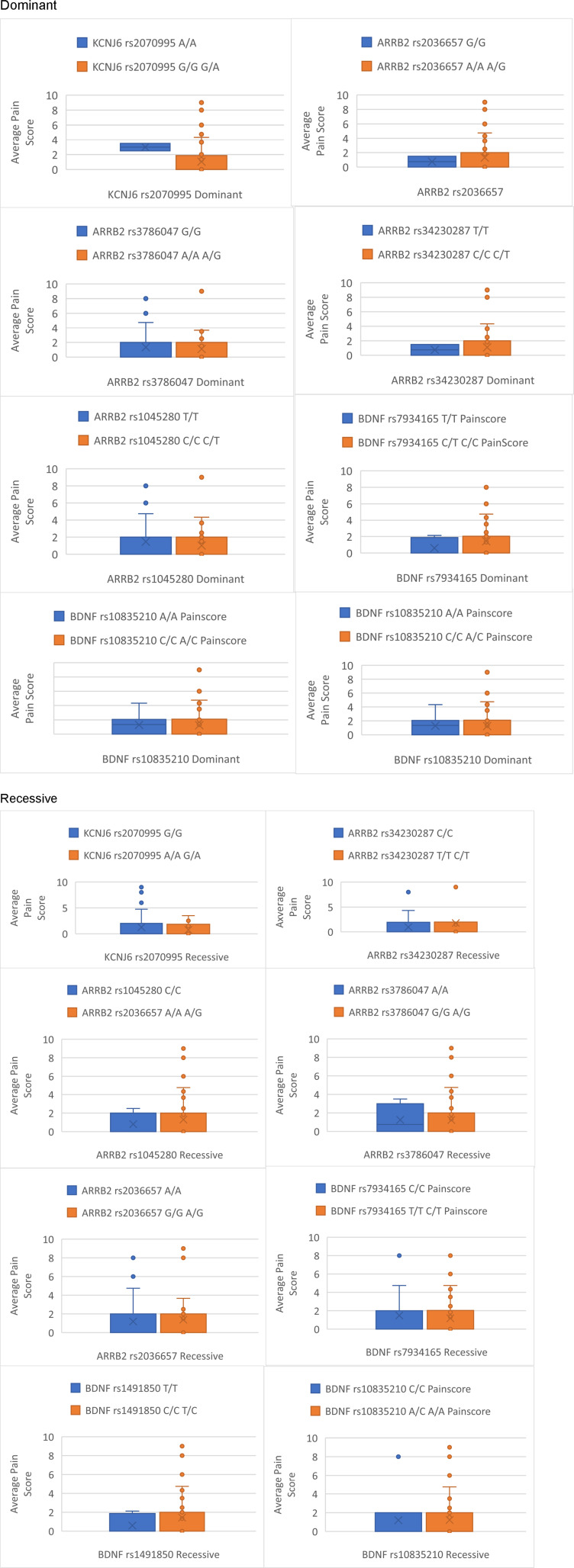
Box Plots Detailing Differences Between Dominant and Recessive Genotypes Average Pain Score

Genotype frequencies for the study population were GG (n = 41), GA (n = 13) and AA (n = 2) and were found to be in HWE (*P* > 0.05). The association of fentanyl dose and pain score are shown for each genotype in Tables 2-5. Figures 2 and 3, detail the average fentanyl dose for genotypes and dominant and recessive representations of alleles in this study. Figures 1 and 4 detail average pain score where they are diagrammatically represented as the data spread for each genotype along with dominant and recessive representations in each patient. The characteristics of association studies of polymorphisms in *KCNJ6* and response to opioids for pain management in advanced cancer, categorized as high/low pain scores or high/low fentanyl dose requirements, are shown in Tables 6 and 7.

**Table 2.** Average Fentanyl Dose for Dominant and Recessive: Test Statistics N (%): Percentage of Test Population for Each Allele, Value: Median Dose Value and Range, H Statistic: Kruskal-Wallace Test Statistic, *P* Value

SNP	Genotype	N (%)	Fentanyl dose (mcg/hr) Median ± IQR (min, max)	H statistic	*P* value
KCNJ6 rs2070995 Dominant	A/A	2 (3.6%)	72.916 ± − (50.0,95.83)	0.138	0.157
	G/A, G/G	54 (96.4%)	41.1 ± 35.5 (12.0,300.0)		
KCNJ6 rs2070995 Recessive	G/G	41 (73.2%)	40.0 ± 44.0 (12.0,300.0)	0.538	0.590
	G/A, A/A	15 (26.8%)	50.0 ± 35.0 (12.0,100.0)		
ARRB2 rs34230287 Dominant	T/T	1 (2.9%)	32.2 Constant	0.138	0.157
	C/T, C/C	34 (97.1%)	38.75 ± 35.75 (12.0,95.83)		
ARRB2 rs34230287 Recessive	C/C	28 (80.0%)	41.75 ± 46.88 (12.0,83.83)	0.112	0.114
	C/T, T/T	7 (20.0%)	32.2 ± 25.87 (12.0, 43.5)		
ARRB2 rs3786047 Dominant	A/A	30 (53.57%)	37.25 ± 30.8 (12.0, 91.0)	1.360	0.174
	G/A, G/G	26 (46.43%)	46.0 ± 43.1 (12.0, 300.0)		
ARRB2 rs3786047 recessive	G/G	4 (7.14%)	34.6 ± 29.7 (12.0, 50.0)	0.315	0.330
	G/A, A/A	52 (92.86%)	43.5 ± 37.38 (12.0,300.00)		
ARRB2 rs1045280 Dominant	C/C	23 (42.59%)	37.0 ± 41.75 (12.0, 91.0)	0.138	0.157
	C/T, T/T	31 (57.41%)	42.2 ± 31.5 (12.0, 300.0)		
ARRB2 rs1045280 recessive	T/T	13 (24.07%)	46.75 ± 56.82 (12.0,300.0)	0.538	0.590
	C/C, C/T	41 (75.93%)	37.5 ± 29.13 (12.0, 91.0)		
ARRB2 rs2036657 Dominant	G/G	2 (4.0%)	22.10 ± − (12.0, 32.2)	0.150	0.180
	G/A, A/A	48 (96.0%)	42.85 ± 36.25 (12.0,300.0)		
ARRB2 rs2036657 recessive	A/A	26 (52.0%)	38.75 ± 41.54 (12.0, 91.0)	0.081	0.088
	G/A, G/G	24 (48.0%)	42.85 ± 45.69 (12.0,300.0)		
BDNF rs10835210 Dominant	A/A	9 (16.1%)	37.5 ± 31.1 (12.0, 87.33)	0.0.739	0.460
	A/C, C/C	47 (83.9%)	43.5 ± 37.5 (12.0, 300.0)		
BDNF rs10835210 recessive	C/C	20 (35.7%)	38.5 ± 47.86 (12.0, 300.0)	0.583	0.560
	A/A, A/C	36 (64.3%)	46.0 ± 34.44 (12.0, 100.0)		
BDNF rs7934165 Dominant	T/T	10 (17.9%)	47.63 ± 31.27 (12.0,100.0)	−0.687	0.492
	C/T, C/C	46 (82.1%)	38.75 ± 37.13 (12.0,300.0)		
BDNF rs7934165 recessive	C/C	18 (32.1%)	37.0 ± 28.04 (12.0, 90.0)	1.153	0.249
	C/T, T/T	38 (67.86%)	48.38 ± 40.63 (12.0,300.0)		
BDNF rs1491850 Dominant	C/C	11(19.64%)	43.5 ± 52.14 (12.0, 300.0)	−0.383	0.702
	C/T, T/T	45 (80.6%)	42.2 ± 34.88 (12.0, 100.0)		
BDNF rs1491850 recessive	T/T	17 (30.4%)	50.0 ± 31.5 (12.0, 75.0)	−0.733	0.463
	C/T, C/C	39 (69.6%)	37.0 ± 35.0 (12.0, 300.0)

**Table 3.** Average Fentanyl dose: Test Statistics N (%): Percentage of Test Population for Each Allele, Value: Median Dose Value and Range, H Statistic: Kruskal-Wallace Test Statistic, *P* Value

SNP	Genotype	N (%)	Fentanyl dose (mcg/hr) Median ± IQR (min, max)	H statistic	*P* value
KCNJ6 rs2070995	A/A	2 (3.6%)	72.9 ± − (50.0, 95.83)	2.208	0.332
	G/A	13 (23.2%)	46.75 ± 30.0 (12.0, 100.0)		
	G/G	41 (73.2%)	40.0 ± 44.0 (12.0, 300.0)		
ARRB2 rs34230287	T/T	1 (2.9%)	32.2 Constant	2.532	0.282
	C/T	6 (17.1%)	34.0 ± 27.28 (12.0, 43.5)		
	C/C	28 (80.0%)	41.75 ± 46.88 (12.0, 95.83)		
ARRB2 rs3786047	A/A	4 (7.14%)	34.6 ± 29.7 (12.0, 50.0)	4.034	0.133
	A/G	22 (39.29%)	48.38 ± 39.5 (12.0, 300.0)		
	G/G	30 (53.57%)	37.25 ± 30.8 (12.0, 91.0)		
ARRB2 rs1045280	C/C	13 (24.07%)	46.75 ± 56.82 (12.0, 300.0)	1.335	0.513
	C/T	18 (33.33%)	38.75 ± 22.44 (12.0, 87.5)		
	T/T	23 (42.59%)	37.0 ± 41.75 (12.0, 91.0)		
ARRB2 rs2036657	G/G	2 (4.0%)	22.1 ± − (12.0, 32.2)	2.527	0.283
	G/A	22 (44.0%)	42.38 ± 50.0 (12.0, 300.0)		
	A/A	26 (52.0%)	38.75 ± 41.54 (12.0, 91.0)		
BDNF rs10835210	A/A	9 (16.1%)	37.5 ± 31.1 (12.0, 87.3)	1.306	0.520
	A/C	27 (48.2%)	50.0 ± 31.5 (12.0, 100.0)		
	C/C	20 (35.7%)	38.5 ± 47.86 (12.0, 300.0)		
BDNF rs7934165	T/T	10 (17.9%)	47.63 ± 31.27 (12.0, 100.0)	1.302	0.522
	C/T	28 (50.0%)	48.38 ± 46.88 (12.0, 300.0)		
	C/C	18 (32.1%)	37.0 ± 28.04 (12.0, 90.0)		
BDNF rs1491850	T/T	11 (19.6%)	43.5 ± 52.14 (12.0, 300.0)	0.971	0.615
	C/T	28 (50.0%)	37.0 ± 25.0 (12.0, 100.0)		
	C/C	17 (30.4%)	50.0 ± 31.5 (12.0, 75.0)

**Table 4.** Average Pain Score for Recessive and Dominant: Test Statistics N (%): Percentage of Test Population for Each Allele, Value: Median Dose Value and Range, H Statistic: Kruskal-Wallace Test Statistic, *P* Value

SNP	Genotype	N (%)	Pain score (0-10) Median ± IQR (min, max)	H statistic	*P* value
KCNJ6 rs2070995 Dominant	A/A	2 (3.6%)	5.42 ± − (1.83, 9.0)	0329	0.375
	G/A, G/G	54 (96.4%)	2.0 ± 2.85 (0.0, 8.0)		
KCNJ6 rs2070995 Recessive	G/G	41 (73.2%)	2.0 ± 3.03 (0.0, 8.0)	−0.074	0.941
	G/A, A/A	15 (26.8%)	2.0 ± 2.333 (0.0, 9.0)		
ARRB2 rs34230287 Dominant	T/T	1 (2.9%)	3.2 Constant	1.213	0.545
	C/T, C/C	34 (97.1%)	1.94 ± 2.48 (0.0, 7.0)		
ARRB2 rs34230287 Recessive	C/C	28 (80.0%)	1.85 ± 2.64 (0.0, 7.0)	2.532	0.282
	C/T, T/T	7 (20.0%)	3.0 ± 2.83 (0.0, 4.0)		
ARRB2 rs3786047 Dominant	A/A	30 (53.57%)	2.0 ± 2.76 (0.0, 7.0)	0.520	0.603
	G/A, G/G	26 (46.43%)	2.3 ± 2.98 (0.0, 9.0)		
ARRB2 rs3786047 recessive	G/G	4 (7.14%)	3.1 ± 6.8 (0.0, 9.0)	0.406	0.432
	G/A, A/A	52 (92.86%)	2.0 ± 2.75 (0.0, 8.0)		
ARRB2 rs1045280 Dominant	C/C	23 (42.59%)	2.0 ± 4.19 (0.0, 7.0)	0329	0.375
	C/T, T/T	31 (57.41%)	2.0 ± 1.7 (0.0, 8.0)		
ARRB2 rs1045280 recessive	T/T	13 (24.07%)	2.5 ± 2.21 (0.0, 8.0)	−0.074	0.941
	C/C, C/T	41 (75.93%)	1.86 ± 2.87 (0.0, 7.0)		
ARRB2 rs2036657 Dominant	G/G	2 (4.0%)	3.1 ± − (3.0, 3.2)	0.367	0.387
	G/A, A/A	48 (96.0%)	2.0 ± 2.75 (0.0, 8.0)		
ARRB2 rs2036657 recessive	A/A	26 (52.0%)	2.0 ± 2.16 (0.0, 7.0)	−1.250	0.211
	G/A, G/G	24 (48.0%)	2.06 ± 3.28 (0.0, 8.0)		
BDNF rs10835210 Dominant	A/A	9 (16.1%)	1.5 ± 1.81 (0.6, 4.33)	0.527	0.598
	A/C, C/C	47 (83.9%)	2.5 ± 3.36 (0.0, 9.0)		
BDNF rs10835210 recessive	C/C	20 (35.7%)	2.3 ± 3.35 (0.6, 4.33)	−0.584	0.559
	A/A, A/C	36 (64.3%)	2.0 ± 2.07 (0.0, 9.0)		
BDNF rs7934165 Dominant	T/T	10 (17.9%)	1.69 ± 1.32 (0.0, 4.0)	−0.203	0.839
	C/T, C/C	46 (82.1%)	2.5 ± 3.3 (0.0, 9.0)		
BDNF rs7934165 recessive	C/C	18 (32.1%)	2.3 ± 3.44 (0.0, 8.0)	−0.203	0.839
	C/T, T/T	38 (67.86%)	2.0 ± 2.48 (0.0, 9.0)		
BDNF rs1491850 Dominant	C/C	11 (19.64%)	2.0 ± − 6.86 (0.0, 8.0)	−0.425	0.671
	C/T, T/T	45 (80.6%)	2.0 ± 2.67 (0.0, 9.0)		
BDNF rs1491850 recessive	T/T	17 (30.4%)	1.88 ± 2.95 (0.0, 9.0)	0.859	0.390
	C/T, C/C	39 (69.6)	2.5 ± 2.5 (0.0, 8.0)

**Table 5.** Average Pain Score: Test Statistics N (%): Percentage of Test Population for Each Allele, Value: Median Dose Value and Range, H Statistic: Kruskal-Wallace Test Statistic, *P* Value

SNP	Genotype	N (%)	Pain score (0-10) Median ± IQR (min, max)	H statistic	*P* value
KCNJ6 rs2070995	A/A	2 (3.6%)	5.42 ± − (1.83, 9.0)	1.117	0.572
	G/A	13 (23.2%)	2.0 ± 2.67 (0.0, 4.75)		
	G/G	41 (73.2%)	2.0 ± 3.03 (0.0, 8.0)		
ARRB2 rs34230287	T/T	1 (2.9%)	3.2 Constant	1.213	0.545
	C/T	6 (17.1%)	2.75 ± 2.98 (0.6, 4.0)		
	C/C	28 (80.0%)	1.85 ± 2.64 (0.0, 7.0)		
ARRB2 rs3786047	A/A	4 (7.14%)	3.1 ± 6.8 (0.0, 9.0)	0.772	0.680
	G/A	22 (39.29%)	2.06 ± 2.98 (0.0, 8.0)		
	G/G	30 (53.57%)	2.0 ± 2.76 (0.0, 7.0)		
ARRB2 rs1045280	C/C	13 (24.07%)	2.5 ± 2.21 (0.0, 8.0)	2.042	0.360
	C/T	18 (33.33%)	1.94 ± 1.98 (0.0, 4.5)		
	T/T	23 (42.59%)	2.0 ± 4.19 (0.0, 7.0)		
ARRB2 rs2036657	G/G	2 (4.0%)	3.1 ± − (3.0, 3.2)	1.422	0.491
	G/A	22 (44.0%)	2.0 ± 3.5 (0.0, 8.0)		
	A/A	26 (52.0%)	2.0 ± 2.16 (0.0, 7.0)		
BDNF rs10835210	A/A	9 (16.1%)	1.5 ± 1.82 (0.6, 4.33)	0.468	0.791
	A/C	27 (48.2%)	2.5 ± 3.2 (0.0, 9.0)		
	C/C	20 (35.7%)	2.3 ± 3.35 (0.0, 8.0)		
BDNF rs7934165	T/T	10 (17.9%)	1.69 ± 1.32 (0.0, 4.0)	1.442	0.486
	C/T	28 (50.0%)	2.5 ± 3.58 (0.0, 9.0)		
	C/C	18 (32.1%)	2.3 ± 3.44 (0.0, 8.0)		
BDNF rs1491850	T/T	11 (19.6%)	2.0 ± 6.86 (0.0, 8.0)	0.971	0.615
	C/T	28 (50.0%)	2.5 ± 2.42 (0.0, 5.5)		
	C/C	17 (30.4%)	1.875 ± 2.95 (0.0, 9.0)

**Table 6.** Relationships Between SNPs and Pain Score (Low Pain Score Less Than or Equal to 3, High Pain Score Greater Than 3)

SNP	Genotype	Low pain score (<3/10) N (%)	High pain score (>3/10) N (%)	χ^2^	*P* value
KCNJ6 rs2070995	A/A	1 (1.8)	1 (1.8)	0.652	0.722
	G/A	10 (17.9)	3 (5.4)		
	G/G	29 (51.8)	12 (21.4)		
ARRB2 rs34230287	T/T	1 (2.9)	0 (0)	3.500	0.174
	C/T	6 (17.1)	0 (0)		
	C/C	18 (51.4)	10 (28.6)		
ARRB2 rs3786047	A/A	4 (7.1)	0 (0)	3.644	0.162
	G/A	13 (23.2)	9 (16.1)		
	G/G	23 (41.1)	7 (12.5)		
ARRB2 rs1045280	C/C	8 (14.8)	5 (9.3)	1.049	0.592
	C/T	14 (25.9)	4 (7.4)		
	T/T	17 (31.5)	6 (11.1)		
ARRB2 rs2036657	G/G	2 (4.0)	0 (0)	0.899	0.638
	G/A	15 (30.0)	7 (14.0)		
	A/A	18 (36.0)	8 (16.0)		
BDNF rs10835210	A/A	7 (12.5)	2 (3.6)	0.213	0.899
	A/C	19 (33.9)	8 (14.3)		
	C/C	14 (25.0)	6 (10.7)		
BDNF rs7934165	T/T	7 (12.5)	3 (5.4)	0.541	0.763
	C/T	19 (33.9)	9 (16.1)		
	C/C	14 (25.0)	4 (7.1)		
BDNF rs1491850	T/T	11 (19.6)	6 (10.7)	1.404	0.496
	C/T	22 (39.3)	6 (10.7)		
	C/C	7 (12.5)	4 (7.1)

**Table 7.** Relationship Between SNPs and Dose (Low Dose Less Than or Equal to 50 mcg/hr, High Dose Greater Than 50 mcg/hr)

SNP	Genotype	Low dose (<50 mcg/hr) N (%)	High dose (>50 mcg/hr) N (%)	χ^2^	*P* value
KCNJ6 rs2070995	A/A	1 (1.8)	1 (1.8)	0.652	0.722
	G/A	10 (17.9)	3 (5.4)		
	G/G	29 (51.8)	12 (21.4)		
ARRB2 rs34230287	T/T	1 (2.9)	0 (0)	3.500	0.174
	C/T	6 (17.1)	0 (0)		
	C/C	18 (51.4)	10 (28.6)		
ARRB2 rs3786047	A/A	4 (7.1)	0 (0)	3.644	0.162
	G/A	13 (23.2)	9 (16.1)		
	G/G	23 (41.1)	7 (12.5)		
ARRB2 rs1045280	C/C	8 (14.8)	5 (9.3)	1.049	0.592
	C/T	14 (25.9)	4 (7.4)		
	T/T	17 (31.5)	6 (11.1)		
ARRB2 rs2036657	G/G	2 (4.0)	0 (0)	0.899	0.638
	G/A	15 (30.0)	7 (14.0)		
	A/A	18 (36.0)	8 (16.0)		
BDNF rs10835210	A/A	7 (12.5)	2 (3.6)	0.213	0.899
	A/C	19 (33.9)	8 (14.3)		
	C/C	14 (25.0)	6 (10.7)		
BDNF rs7934165	T/T	7 (12.5)	3 (5.4)	0.541	0.763
	C/T	19 (33.9)	9 (16.1)		
	C/C	14 (25.0)	4 (7.1)		
BDNF rs1491850	T/T	11 (19.6)	6 (10.7)	1.404	0.496
	C/T	22 (39.3)	6 (10.7)		
	C/C	7 (12.5)	4 (7.1)		

### Association of Polymorphisms in BDNF and Fentanyl Response

Genotype frequencies for our study population were as follows: rs34230287 CC (n = 31), CT (n = 15), TT (n = 0); rs3786047 AA (n = 2), AG (n = 19), GG (n = 25); rs1045280 CC (n = 12), CT (n = 9), TT (n = 25); rs2036657 GG (n = 2), GA (n = 19), AA (n = 25). All genotypes were in HWE (*P* > 0.05). The association of fentanyl dose and pain score are shown for each genotype in Tables 2-5. The data spread for each genotype and dominant and recessive alleles is represented in Figures 2 and 3, detailing the average fentanyl dose for genotypes and dominant and recessive representations of alleles, whilst Figure 1 and 4 detail average pain scores are diagrammatically represented. The characteristics of association studies of polymorphisms in *BDNF* and response to opioids for pain management in advanced cancer, categorized as high/low pain scores or high/low fentanyl dose requirements, are shown in Tables 6 and 7.

### Association of Polymorphisms in ARRB2 and Fentanyl Response

Genotype frequencies for our study population were as follows: rs34230287 CC (n = 31), CT (n = 15), TT (n = 0); rs3786047 AA (n = 2), AG (n = 19), GG (n = 25); rs1045280 CC (n = 12), CT (n = 9), TT (n = 25); rs2036657 GG (n = 2), GA (n = 19), AA (n = 25). All genotypes were in HWE (*P* > 0.05). The association of fentanyl dose and pain score are shown for each genotype in Tables 2-5. Diagrammatically the data spread for each genotype, and dominant and recessive alleles are represented in Figures 2 and 3 for average fentanyl dose, whilst Figures 1 and 4 detail the average pain score. The characteristics of association studies of polymorphisms in *ARRB2* and response to opioids for pain management in advanced cancer, categorized as high/low pain scores or high/low fentanyl dose requirements, are again shown in Tables 6 and 7.

## Discussion

The study had three key aims: (1) To assess if patient variables influenced fentanyl dosage and pain score; (2) To investigate if SNPs in *ARRB2*, *KCNJ6*, and *BDNF* affected pain scores; and (3) To examine if SNPs in *ARRB2*, *KCNJ6*, and *BDNF* relate to fentanyl dosage. The study recruited 56 adult in-patients and outpatients of an oncology and palliative care service with advanced cancer for a pharmacogenomic sub-study of an open-label fentanyl dose individualization trial. Regression analysis examined the dependence of outcomes on non-genetic patient characteristics, while the Kruskal-Wallis H-test evaluated the association between genotypes and fentanyl dose or pain score. Low (≤3/10) or high (>3/10) pain scores and low (≤50 mcg/h) or high (>50 mcg/h) fentanyl doses were categorized to identify significant associations. The study found no link between the *KCNJ6* gene’s rs2070995, *ARRB2*, or BNF SNPs and fentanyl dose requirements or pain scores, achieving its first objective. No dosage or pain score differences were observed between different genotypes, regardless of dominant or recessive alleles. The second objective showed no association between the investigated SNPs with pain score, while the third objective found no association between the SNPs examined in the study with fentanyl dose.

The study looked at four specific genes and examined their relationship to pain and fentanyl dosage. However, no significant results were found to support the hypothesis that these genes determine fentanyl dosage. Box and whisker plots were created to compare each gene to average dose and pain score, however, the data was skewed due to the small sample size. A larger population may be needed for a more detailed examination of this type of population. Nevertheless, as the study also identified that the current population represented a diverse population, increasing the sample size may not yield different results.

### The Associated Outcomes of KCNJ6

Previous studies investigating the *KCNJ6* gene have yielded inconsistent findings. Some studies found no association between the gene and factors such as medication dosage or pain control.^23,24^ In contrast, significant differences in outcomes have been identified depending on the different SNPs present within *KCNJ6.*^6^ These studies included the examination of patients with different types of pain and treatment with various opioids. At the time of this study, data specifically focused on the use of fentanyl as a single intervention involving the SNPs in this study has not been investigated.^25^

### The Associated Outcomes of ARRB2

Studies on the *ARRB2* gene have shown varying outcomes. Some studies suggest that multiple SNP interactions may cause significant association with methadone dosage.^26^ One study found no associations with fentanyl or oxycodone.^27,28^ Another study found that rs1045280 may influence opioid dosing requirements, while others did not find any significant association between this SNP and opioid dose.^9,23,29,30^ These studies involved patients with different types of pain and different opioids, with limited investigations into the role of varying SNPs in *KCNJ6* outside of ARRB2 SNPs, rs1045280 and rs3786047.

### The Associated Outcomes of BDNF

Numerous studies in recent years have focused on mental health, overdose, and suicide in relation to *BDNF*. However, there is growing research on the role of *BDNF* in opioid use for cancer and pain. Some studies found that SNPs in *BDNF* increase opioid demand due to an altered response,^31,32^ while others found no association between genetic factors and pain control or opioid adverse events.^28,33^ This study aimed to fill in gaps in understanding the role of *BDNF* regarding fentanyl dosing requirements regarding SNPs rs7934165, rs10835210, rs1491850 and the use of fentanyl.

### Limitations of the Study

Conducting studies on cancer management can be challenging due to various factors, including the individualized nature of dose titration while taking into consideration breakthrough and/ or background opioids. Patients may be ill due to various cancers or drop out of the study due to death or sickness, resulting in small sample sizes and the need to collect data over an extended period. In this study, data was collected over a period of two years, from 2013 to 2015. Another limitation is the mixed lineage of the Australian population, making it challenging to group participants into distinct homogenous groups based on race or ethnicity. Monitoring the dose increases in maintenance therapy can help gauge patient opioid demand. Pain relief is the core role of pain management in palliative care, and tools including pain catastrophizing are not critical in such circumstances and were not used in the study.

## Conclusion

The study examined whether patient characteristics may be linked to fentanyl dose requirements and pain scores, with no significant associations found. Moreover, there was no link between the SNPs examined in *KCNJ6*, *ARRB2*, and *BDNF* with high/low pain scores or high/low fentanyl dose requirements in this study. However, these findings may not apply to larger studies due to the small sample size (n = 56). Furthermore, more research is needed to determine whether these SNPs may be used for genetic testing to determine opioid dosing. The study did not find evidence linking the SNPs examined in *KCNJ6*, *ARRB2*, and *BDNF* to sensitivity to fentanyl dosing or pain scores, with larger studies required to determine this for the general population. The study also did not show that the polymorphisms in *ARRB2* may be linked to greater responses to opioids, which could be due to a lack of correlation between the SNPs analyzed and other genetic factors influencing the correlation between dose and response. Although the study did not find evidence that *BDNF* influenced the reliance/addiction characteristics of the study cohort, further research may uncover more significant findings regarding dominant alleles of these SNPs in larger populations.^34^ Our study confirmed no association between polymorphisms in *ARRB2* and clinical response to transdermal fentanyl for pain management in advanced cancer. However, it may provide insight into the impact of genetics on the pharmacological effects of medications, particularly in advanced cancer pain. Limitations in participant retention and the need for long-term studies on this population continue to hinder research in this area.

## Data Availability

All data generated or analyzed during this study are included in this published article and/or its supplementary information files. All genotype data has been deidentified to ensure no connection to individual participants.*

## Abbreviations

ARRB2: β-arrestin2
BDNF: Brain-derived neurotrophic factor
BMI: Body mass index
CPIC: Clinical Pharmacogenetics Implementation Consortium
gDNA: genomic DNA
GPCR: G-protein-coupled receptor
HWE: Hardy-Weinberg equilibrium
IQR: Interquartile range
MAF: Minor allele frequency
SD: Standard deviation
SNP: Single Nucleotide Polymorphism
